# Investigation of the interaction between genetic risk score (GRS) and fatty acid quality indices on metabolic syndrome among overweight and obese women

**DOI:** 10.1186/s12920-024-01838-2

**Published:** 2024-04-29

**Authors:** Niloufar Rasaei, Elnaz Daneshzad, Alireza Khadem, Fatemeh Gholami, Mahsa Samadi, Khadijeh Mirzaei

**Affiliations:** 1 Department of Community Nutrition, School of Nutritional Sciences and Dietetics, Tehran University of Medical Sciences (TUMS), Tehran, P. O. Box: 14155-6117, Iran; 2 Non-Communicable Diseases Research Center, Alborz University of Medical Sciences, Karaj, Iran; 3 Department of Nutrition, Science and Research Branch, Islamic Azad University, Tehran, Iran

**Keywords:** Genetic risk score, Fatty acid quality index, Metabolic syndrome, Overweight, Obesity

## Abstract

**Background and aim:**

Metabolic syndrome is one of the major public-health challenges, affecting one-quarter of the world population. Fatty acid quality indices are novel determinants of this disease and their interactions with genetic factors may have an impact on metabolic syndrome risk. Therefore, we aimed to investigate the interaction between genetic risk score (GRS) and fatty acid quality indices with metabolic syndrome (MetS) among overweight and obese women.

**Methods:**

In the present cross-sectional study, 279 overweight and obese women (18–48 years old) were included. Several anthropometric measurements such as weight, height, body mass index (BMI), waist circumference (WC), and body fat percent (BF%) were measured. Also, systolic and diastolic blood pressure (SBP and DBP) were measured. Biochemical determination was performed for fasting blood glucose (FBS), triglyceride (TG), and high-density lipoprotein (HDL). MetS was determined according to National Cholesterol Education Program (NCEP ATP III) criteria. Dietary intake was evaluated by a validated and reliable 147-item semi-quantitative food frequency questionnaire. Cholesterol-saturated fat index (CSI) and the ratio of omega-6/omega-3 (ω-6/ω-3) essential fatty acids were considered as fat quality indices. The salting-out method was used to extract the total DNA. The unweighted GRS was calculated using the risk alleles of the three single nucleotide polymorphisms. The total average GRS value was 2 and the sum of the risk alleles of the 3 polymorphisms was 6.

**Result:**

The results of our analysis showed that after controlling for age, energy intake, BMI, and physical activity, there was a positive interaction between T2 of GRS and T2 of N6/N3 ratio on WC (β = 7.95, 95%CI = 0.83,15.08, *P* = 0.029), T3 of GRS and T2 of N6/N3 ratio on DBP (β = 5.93, 95%CI= -0.76,12.63, *P* = 0.083), and FBS (β = 6.47, 95%CI = 0.59,13.53, *P* = 0.073), T3 of GRS and T3 of N6/N3 ratio on TG (β = 54.42, 95%CI = 1.76,107.08, *P* = 0.043), and T3 of GRS and T3 of CSI on BF% (β = 3.55, 95%CI= -0.35,7.45, *P* = 0.075). Also T2 of GRS in the interaction with T3 of CSI leads to an decrease − 8.35 mg/dl in HDL level after adjustment in (β= -8.35, 95%CI= -17.34,0.62, *P* = 0.068).

**Conclusion:**

It seems the interaction of GRS and fatty acid quality indices is positively associated with several components of metabolic syndrome such as WC, TG and BF%. Our findings are of importance to public health, considering the high consumption of foods that are high on fatty acids. Conflicting evidence of many previous studies regarding the effect of fat intake and obesity and cardiovascular diseases could be because of the gene-diet interactions and genetic heterogeneity across various ethnic groups. Hence, the synergism effect of genetic and dietay intakes should be considered in future studies.

**Supplementary Information:**

The online version contains supplementary material available at 10.1186/s12920-024-01838-2.

## Introduction

Metabolic syndrome (MetS) is a cluster of components including obesity, hypertriglyceridemia, high-density lipoprotein cholesterol (HDL), hypertension, and high fasting blood glucose [1], which is associated with an increased risk of type 2 diabetes mellitus (T2DM) and cardiovascular disease (CVD) [2]. Given that 20–25% of adults worldwide have MetS, it is regarded as one of the major contributors to serious global health challenges over the current century [3, 4]. Furthermore, the prevalence of this syndrome in Asian countries varies, ranging from 10 to 20% [5] with an estimated 8–35% of the Iranian population affected [6–9].

As a multifactorial disease, environmental conditions such as dietary intake and genetic variations are involved in its pathogenesis [10–15]. Apart from a positive connection between total fat intake and risk of MetS, as reported in a Japanese-Brazilian population, it is crucial to mention that dietary fat quality is also of utmost importance [16]. During the preceding decade, Connor et al. proposed the Cholesterol-Saturated Fat Index (CSI), a novel index of dietary fat quality [17]. Alongside CSI, Simopoulos indicated the importance of the omega-6/omega-3 essential fatty acids (EFA) ratio [18]. The CSI is a dietary self-monitoring tool that reflects the cholesterol and saturated fat content of food and helps patients to improve their cholesterol-lowering eating plan, by its influence on self-management and better food selection. As a matter of fact, lower CSI represents reduced saturated fatty acid (SFA) and cholesterol [19]. Studies have reported that following dietary pattern high in SFA might be attributed to weight gain and elevated risk of metabolic disturbances [20, 21]. Moreover, polyunsaturated fatty acids such as linoleic acid have shown a tendency to attenuate the risk of MetS due to their relation with insulin resistance [16]. In this regard, a balanced ratio of omega-6/omega-3 EFA plays a central role in the prevention and management of chronic diseases [18]. Of note is that, genetic predisposition has been recognized as a significant risk factor for MetS [22] and genetic risk score (GRS), calculated through the summing of risk alleles for each single nucleotide polymorphisms (SNP) [23], was developed to determine the association between MetS and genetic factors. Here, large-scale genome-wide association studies (GWAS) identified obesity-related SNPs for three novel genes of Melanocortin-4 Receptor (MC4R), Caveolin (CAV), and Cryptochrome (CRY) [24–26]. Following this identification, the “gene-environment interaction” hypothesis was suggested [27]. In accordance with this hypothesis, individuals with adherence to a western dietary pattern that is high in saturated fat and low in linoleic acid, indicated an increased risk for MetS, considering genetic predisposition [28]. However, to the author’s knowledge, no literature has been generated on the interaction between BMI-GRS, based on aforementioned genetic variants, and dietary fat quality indices on MetS thus far, and most of them evaluated single SNPs interactions [29]. Therefore, this study aimed to investigate the interaction between BMI-GRS including MC4R (rs17782313), CAV-1 (rs3807992), and Cry-1 (rs2287161) with dietary fat quality indices according to CSI and omega-6/omega-3 EFA ratio on MetS in overweight and obese women.

## Method and materials

### Study population

In the present cross-sectional study, among all health centers of Tehran University of Medical Sciences, 20 health centers were selected randomly in 2018. Through multi-stages simple random sampling, 279 overweight and obese women who were referred to one of those health centers were entered. Participants (overweight and obese) with BMI of 25 to 40 kg/m2 [30] and ages range of 18 to 48 years were included. All subjects signed the written informed consent at begin of the study and the Tehran University of Medical Sciences (TUMS) approved them. Exclusion criteria were as follows: patients with malignancies; liver, kidney, or cardiovascular diseases; all types of diabetes; thyroid disease; any other acute and chronic diseases, menopause or pregnant women, lactation, weight loss supplementation, antihypertensive or lowering glucose and lipid medications, dieting during the last year, and smoking. The present study was approved by The Ethics Committee of the TUMS (assigned number: IR.TUMS.VCR.REC.1398.636). Regarding the following formula, sample size of 279 was estimated to sufficiently evaluate the outcomes (both primary and secondary) and achieve r = 0.25 [31], β = 0.95, and a type I error α = 0.05. Formula: n= (([Z1−α+Z1−β) ×<![CDATA[ \surd ]]>1-r2]/r) 2+2 [32].

### Anthropometric and blood pressure assessment

Several anthropometric measurements were measured by bioelectrical impedance analyzer BIA, including weight, body mass index (BMI), and body fat percent (BF%); following the manufacturer’s protocol (InBody 770 scanner from InBody Co. (Seoul, Korea)) [33]. Subjects were required to remove extra clothing and metal objects such as rings, earrings, watches, sweaters, coats, and shoes.

Moreover, the height was measured using a non-stretch tape measure in a standing up position with 0.5 cm precision. Waist circumference (WC) was measured using the most prominent portion and the narrowest portion respectively with 0.5 cm precision.

Blood pressure was measured using an appropriate cuff according to arm size. It was measured for two times after 5 min of rest. Finally, the average of two measurements was recorded.

### Physical activity assessment

Physical activity (PA) was assessed based on the validated and reliable self-report instrument called the short-form of the International Physical Activity Questionnaire (IPAQ). The IPAQ assesses the duration and frequency of typical daily activities throughout a week in the preceding year. It quantifies the participants’ weekly physical activity levels in metabolic equivalent hours (MET-h/week) [34].

### Biochemical and hormonal determination

Venous blood was collected between 8:00 to 10:00 a.m. after fasting overnight. Serum samples were centrifuged, stored at − 80 °C, and analyzed by using a single assay technique. Fasting blood glucose (FBS), and triglyceride (TG) were measured by using glucose oxidase-phenol 4-aminoantipyrine peroxidase (GOD-PAP) and glycerol-3-phosphate oxidase–phenol 4-aminoantipyrine peroxidase (GPOPAP) enzymatic endpoint, respectively. We measured high-density lipoprotein (HDL) cholesterol using by direct enzymatic clearance assay. Randox Laboratories (Hitachi 902) kit was used for all measurements.

All samples were assessed by standard methods at the Nutrition and Biochemistry Laboratory of the School of Nutritional and Dietetics at TUMS.

### Assessment of metabolic syndrome (MetS)

MetS was determined according to National Cholesterol Education Program (NCEP ATP III) criteria [35]. Presence of 3 or more of the following criteria was considered as MetS: (1) abdominal obesity [≥ 102 cm for men and WC ≥ 88 cm for women]; (2) hypertriglyceridemia [≥ 150 mg/dL]; (3) reduced HDL [< 40 mg/dL for men and < 50 mg/dL for women]; (4) raised FBS [FBS > 100 mg/dL]; and (5) raised blood pressure [systolic blood pressure ≥ 130 mmHg and/or diastolic blood pressure ≥ 85 mm Hg].

### Dietary intake assessment

Dietary intake was evaluated by a validated and reliable 147-item semi-quantitative food frequency questionnaire (FFQ) [36]. Participants recorded their usual diet consumption frequency through a day, week, or month in the last year in the presence of a dietitian. Dietary intake was analyzed for energy intake, macronutrients, and micronutrients utilizing the NUTRITIONIST 4 (First Data Bank, San Bruno, CA) food analyzer [37].

### Dietary fat quality indices

FFQ was evaluated to determine those food items to be included. Cholesterol-saturated fat index (CSI) and the ratio of omega-6/omega-3 (ω-6/ω-3) essential fatty acids were considered as fat quality indices. CSI indicates the concentrations of cholesterol and saturated fat in foods. By dividing cholesterol by saturated fat content of food items that were derived from FFQ, CSI was presented [19]. A low CSI represents low cholesterol and/or saturated fat content, therefore a diet with lower CSI has hypocholesterolemic and low atherogenic potential. Also, the ratio of ω-6 to ω-3 was calculated according to dividing ω-6 to ω-3 contents of food items which had been evaluated by FFQ [17, 18].

FFQ was evaluated to determine those food items to be included. Cholesterol-saturated fat index (CSI) and the ratio of omega-6/omega-3 (ω-6/ω-3) essential fatty acids were considered as fat quality indices. CSI indicates the concentrations of cholesterol and saturated fat in foods. By dividing cholesterol by saturated fat content of food items that were derived from FFQ, CSI was presented [19]. A low CSI represents low cholesterol and/or saturated fat content, therefore a diet with lower CSI has hypocholesterolemic and low atherogenic potential. Also, the ratio of ω-6 to ω-3 was calculated according to dividing ω-6 to ω-3 contents of food items which had been evaluated by FFQ [17, 18].

### Genotyping and GRS

The salting-out method was used to extract the total DNA [38]. 1% agarose gel was used to assess the DNA integrity and a nanodrop 8000 Spectrophotometer (Thermo Scientific, Waltham, MA, USA) was used to assess DNA concentration. SNP genotyping was carried out using the TaqMan Open Array (Life Technologies Corporation, Carlsbad, CA, USA) [39].

The CAV-1 (rs3807992) forward primer is 3′AGTATTGACCTGATTTGCCATG 5′ and the reverse primer is 5′ GTCTTCTGGAAAAAGCACATGA 3′. The fragments containing three genotypes were distinguished: GG, AA, and GA. The Cry1 (rs2287161) forward primer is 5′-GGAACAGTGATTGGCTCTATCT − 3′ and the reverse primer is 5′-GGTCCTCGGTCTCAAGAAG-3′. Then, the fragments containing three genotypes were distinguished: CC, GG, and GC. The MC4R gene primer was selected based on a previous study [40]. The MC4R (rs17782313) forward primer is 5- AAGTTCTACCTACCATGTTCTTGG-3 and the reverse primer is 5-TTCCCCCTGAAGCTTTTCTTGTCATTTTGAT-3. Then, fragments containing three genotypes were distinguished: CC, TT, and CT. We created the GRS by combining three single nucleotide polymorphisms [CAV-1 (rs3807992), Cry-1 (rs2287161), and MC4R (rs17782313)] that had previously been linked to obesity-related traits in GWAS and other studies [26, 41, 42]. The risk alleles for higher BMI were assigned to each SNP by recoding them into 0, 1, or 2. The unweighted GRS was calculated using the risk alleles of the three SNPs. Higher scores indicate greater genetic susceptibility to higher BMI on the GRS scale, which ranges from 0 to 6 [43].

### Statistical analyses

The normal distribution of data was assessed by the Kolmogorov-Smirnov test. General characteristics of participants were presented as mean ± standard deviation, minimum and maximum. Analysis of variance (ANOVA) and analysis of covariance (ANCOVA) were conducted to compare anthropometric indices, blood pressure, FBS, and lipid profile among participants. A generalized linear model (GLM) was used in crude and adjusted models to evaluate the associations of MetS components (dependent variable) and GRS (independent variable). Adjustments were performed for age, energy intake, PA, and BMI. All statistical analysis was performed using SPSS version 23.0 (SPSS, Chicago, IL, USA). A P-value lower than 0.05 was considered statistically significant and a P-value lower than 0.1 was considered marginally significant.

## Result

### Study population characteristics

A total of 279 overweight and obese women were evaluated in this study. The mean height, weight, BMI and WC of participants were 161.28 cm, 80.75 kg, and 31.03 kg/m^2^ and 99.22 cm respectively. Also, the mean of metabolic factors including FBS, TG, and HDL of participants were 87.26 mg/dl, 120.80 mg/dl, and 46.45 mg/dl, respectively.

### Mean and standard deviation (SD) of general characteristics according to tertiles of CSI and N6/N3

The general characteristics of study participants among tertiles of the CSI and N6/N3 ratio were presented in Table 1. According to this table, p-values for all variables were reported in the crude and adjusted model after controlling for potentially confounding variables (age, energy intake, physical activity, and BMI). In the crude model, a significant mean difference was observed among tertiles of the CSI in terms of age (*P* = 0.003), and TG (*P* = 0.010), while none of the variables were significant among tertiles of the N6/N3. After adjustment with potential cofounders, the mean difference of age (*P* = 0.021) and TG (*P* = 0.020) remained significant and the PA (*P* = 0.048) of participants among tertiles of the CSI became significant, while no significant difference was observed in any of the variables among tertiles of the N6/N3 ratio (*P* > 0.05). BMI was considered as collinear for anthropometrics variables.

**Table 1:** Mean and SD of general characteristics according to tertiles of CSI and N6/N3 in obese and overweight women (*n* = 279)

Variables†	CSI				
	Mean ± SD			P-value	P-value ^a^
	T_1_ (*n* = 78)	T_2_ (*n* = 79)	T_3_ (*n* = 78)		
Age (years)	33.75 ± 8.71	37.15 ± 7.37	38.11 ± 8.61	**0.003**	**0.021**
PA (MET-min/week)	834.51 ± 830.12	1106.28 ± 1372.74	1040.69 ± 1106.06	0.340	**0.048**
**Anthropometric measurements**					
Weight (kg)	78.04 ± 10.52	80.18 ± 10.80	79.03 ± 9.47	0.427	0.436
Height (cm)	162.04 ± 5.63	160.90 ± 5.13	160.66 ± 6.16	0.266	0.543
WC (cm)	96.84 ± 9.43	98.50 ± 9.18	97.96 ± 8.35	0.502	0.542
BMI (kg/)	29.83 ± 3.65	30.89 ± 3.57	30.58 ± 3.36	0.157	0.496
BF (%)	39.75 ± 6.40	41.63 ± 4.19	41.37 ± 4.75	0.052	0.13
**Blood pressure**					
SBP (mmHg)	110.02 ± 12.44	112.64 ± 14.54	111.77 ± 14.48	0.495	0.656
DBP (mmHg)	76.71 ± 10.47	78.12 ± 9.31	78.22 ± 9.91	0.577	0.736
**Metabolic factors**					
FBS (mg/dl)	85.05 ± 8.26	87.75 ± 8.31	88.94 ± 11.71	0.035	0.365
TG (mg/dl)	102.37 ± 50.54	125.54 ± 74.63	135.41 ± 77.74	**0.010**	**0.020**
HDL (mg/dl)	45.47 ± 7.91	46.96 ± 11.90	47.62 ± 11.54	0.432	0.896
**Variables**†	**N6/N3**				
	**Mean ± SD**			**P-value**	**P-value**_**a**_
	**T**_**1**_ **(** ***n*** ** = 93)**	**T**_**2**_ **(** ***n*** ** = 93)**	**T**_**3**_ **(** ***n*** ** = 93)**		
Age (years)	35.95 ± 8.20	36.08 ± 8.45	37.40 ± 8.72	0.434	0.454
PA (MET-min/week)	960.36 ± 926.07	1192.29 ± 1445.85	812.75 ± 727.60	0.082	0.165
**Anthropometric measurements**					
Weight (kg)	81.12 ± 10.74	80.84 ± 11.89	78.01 ± 9.77	0.098	0.554
Height (cm)	162.02 ± 5.47	161.79 ± 5.77	160.15 ± 6.09	0.058	0.876
WC (cm)	98.81 ± 9.13	99.62 ± 10.11	96.79 ± 8.49	0.103	0.253
BMI (kg/)	30.90 ± 3.93	30.91 ± 3.63	30.37 ± 3.61	0.532	0.576
BF (%)	41.20 ± 5.88	41.05 ± 5.15	41.55 ± 4.91	0.809	0.985
**Blood pressure**					
SBP (mmHg)	110.35 ± 14.18	112.51 ± 12.88	110.59 ± 13.55	0.503	0.300
DBP (mmHg)	76.94 ± 10.37	78.08 ± 9.42	77.62 ± 9.10	0.727	0.259
**Metabolic factors**					
FBS (mg/dl)	87.06 ± 9.31	86.35 ± 9.12	88.22 ± 10.43	0.468	0.061
TG (mg/dl)	118.33 ± 67.37	121.08 ± 72.83	123.72 ± 70.10	0.888	0.307
HDL (mg/dl)	46.18 ± 10.16	47.51 ± 11.04	46.42 ± 10.70	0.716	0.740

BF%; body fat percentage; BMI: body mass index; CSI: cholesterol to saturated fat index; DBP: diastolic blood pressure; FBS: fasting blood sugar; HDL: high density lipoprotein; PA: physical activity; SD: standard deviation; SBP: systolic blood pressure; T: tertile; TG: triglyceride; WC: waist circumference.

† Calculated by analysis of variance (ANOVA)

a: Adjusted for age, BMI, physical activity, and total energy intake.

*p* < 0.05 was considered significant

### Mean and SD of general characteristics according to tertiles of GRS

**Fig. 1:**
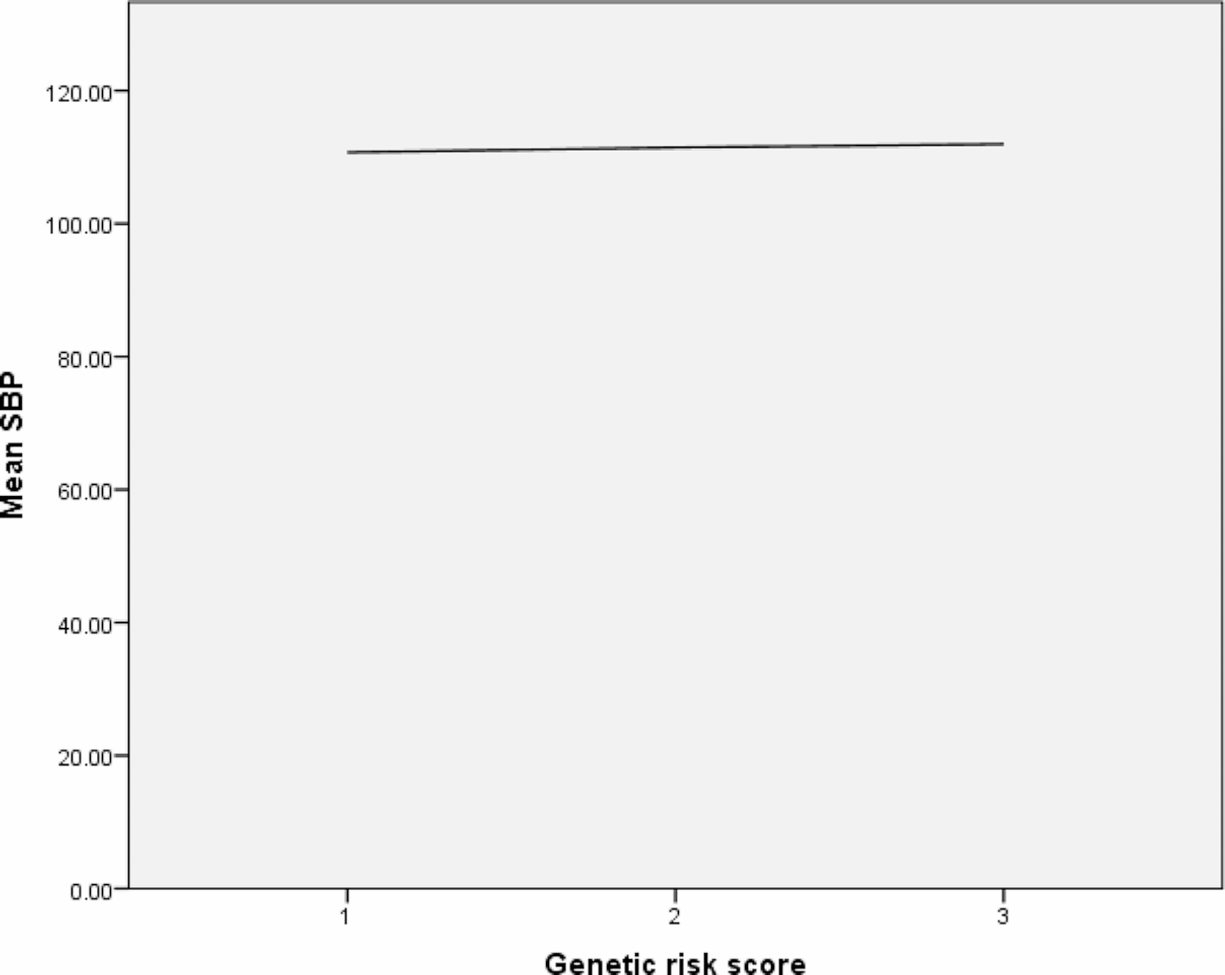
Mean and SD of SBP according to tertiles of GRS

**Fig. 2:**
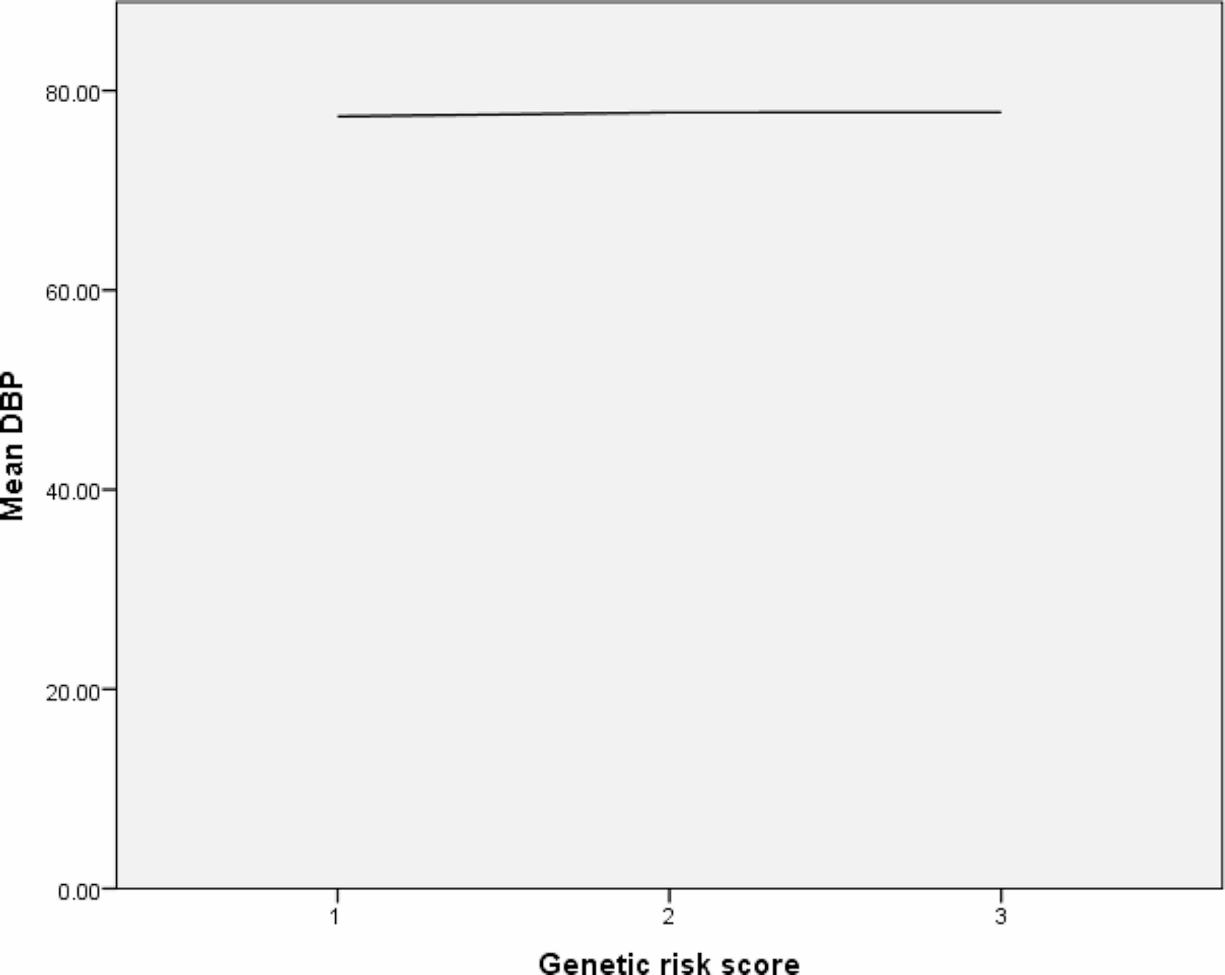
Mean and SD of DBP according to tertiles of GRS

**Fig. 3:**
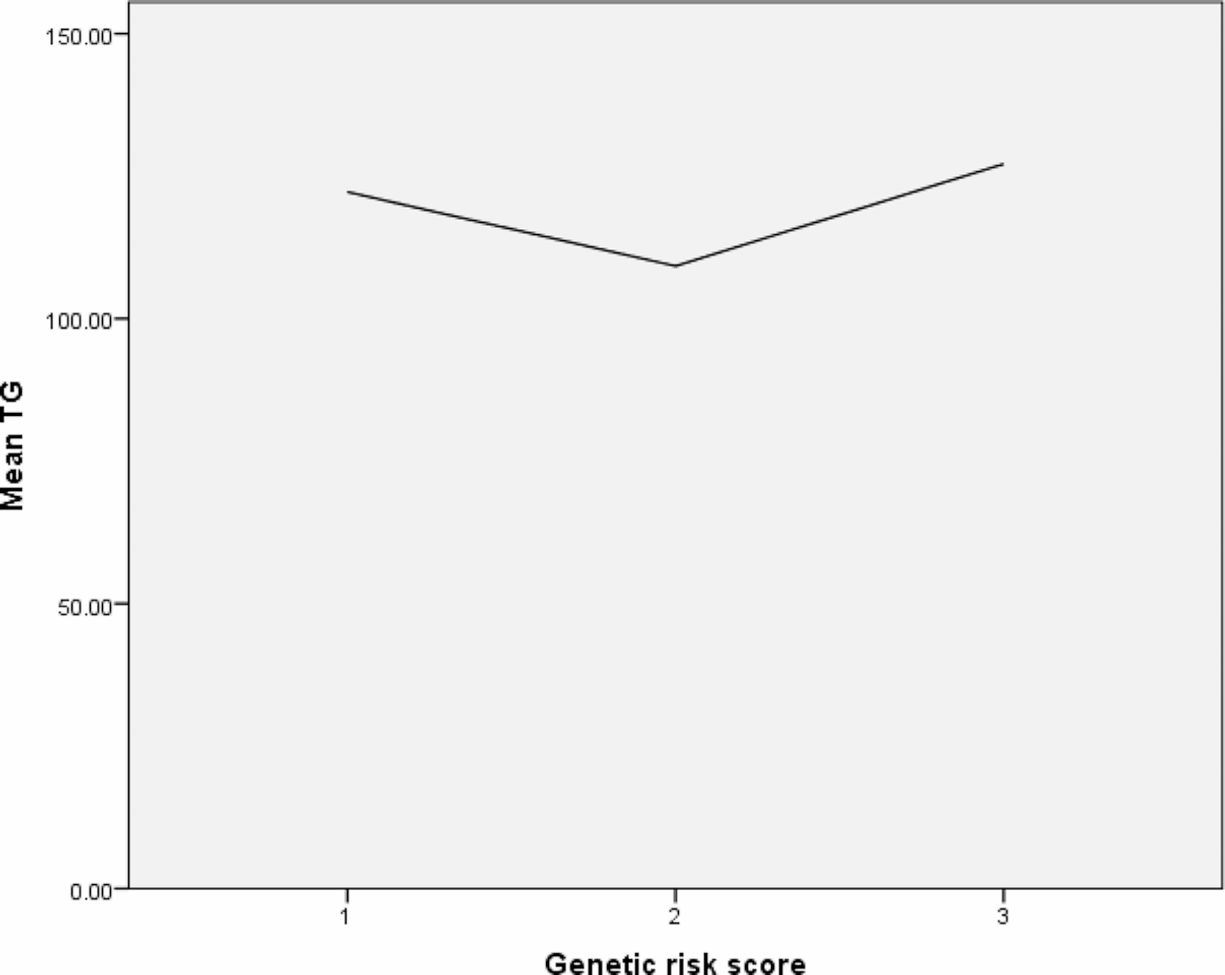
Mean and SD of TG according to tertiles of GRS

**Fig. 4:**
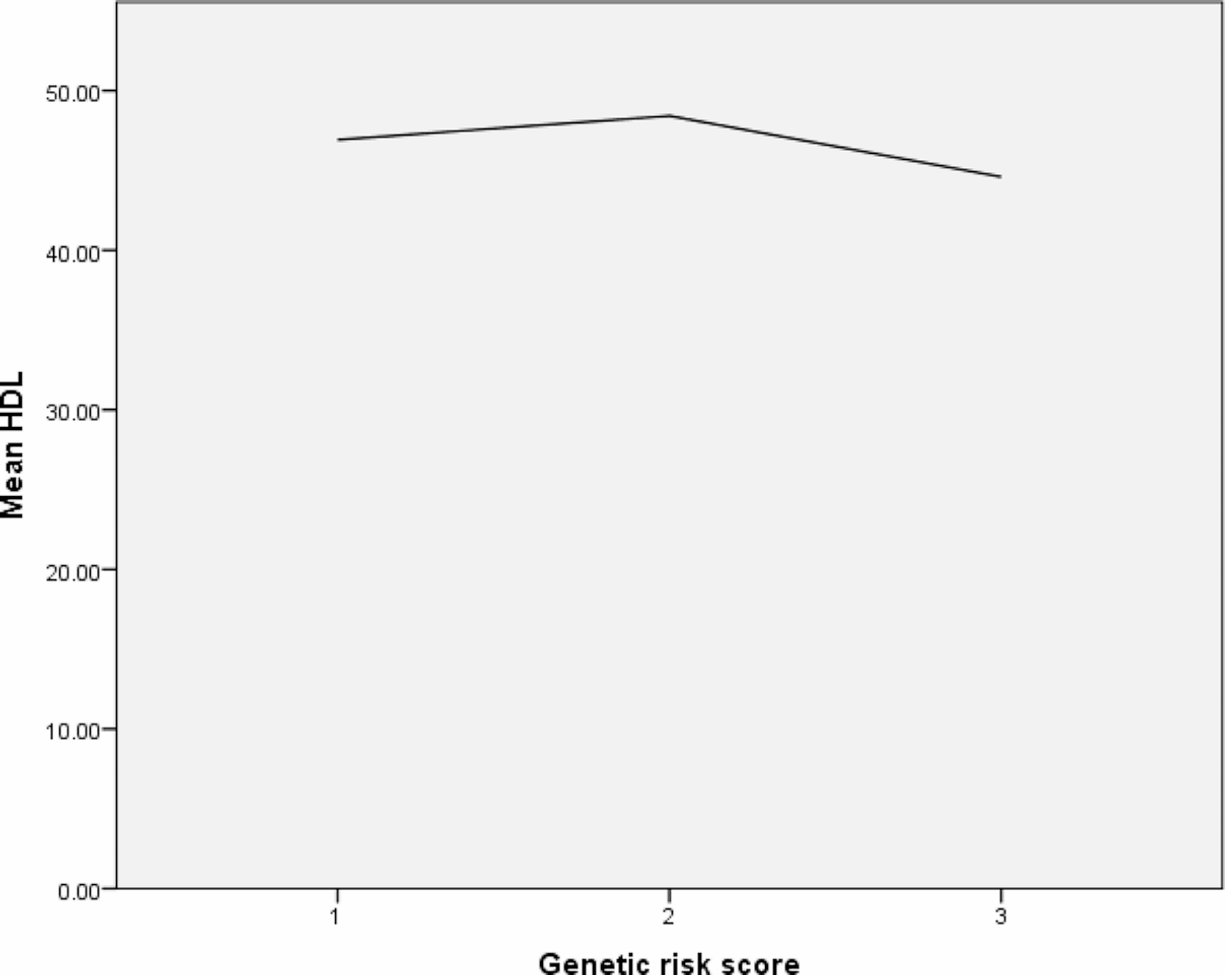
Mean and SD of HDL according to tertiles of GRS

**Fig. 5:**
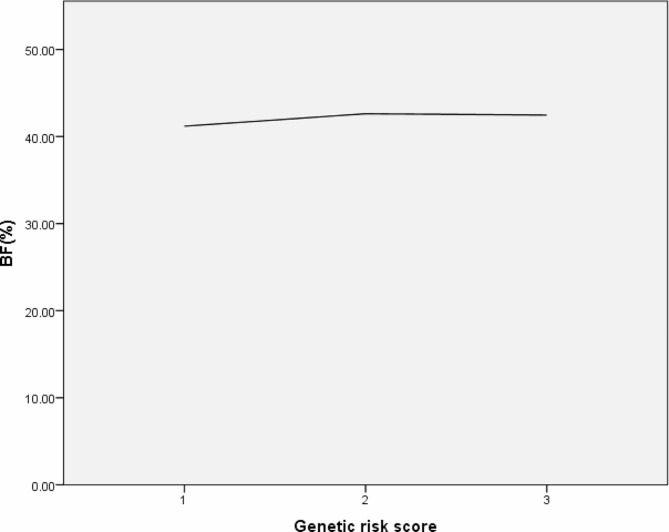
Mean and SD of BF% according to tertiles of GRS

**Fig. 6:**
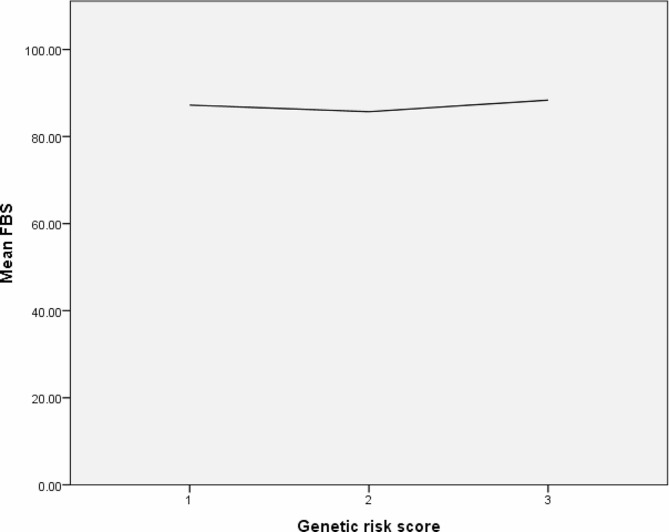
Mean and SD of FBS according to tertiles of GRS

**Fig. 7:**
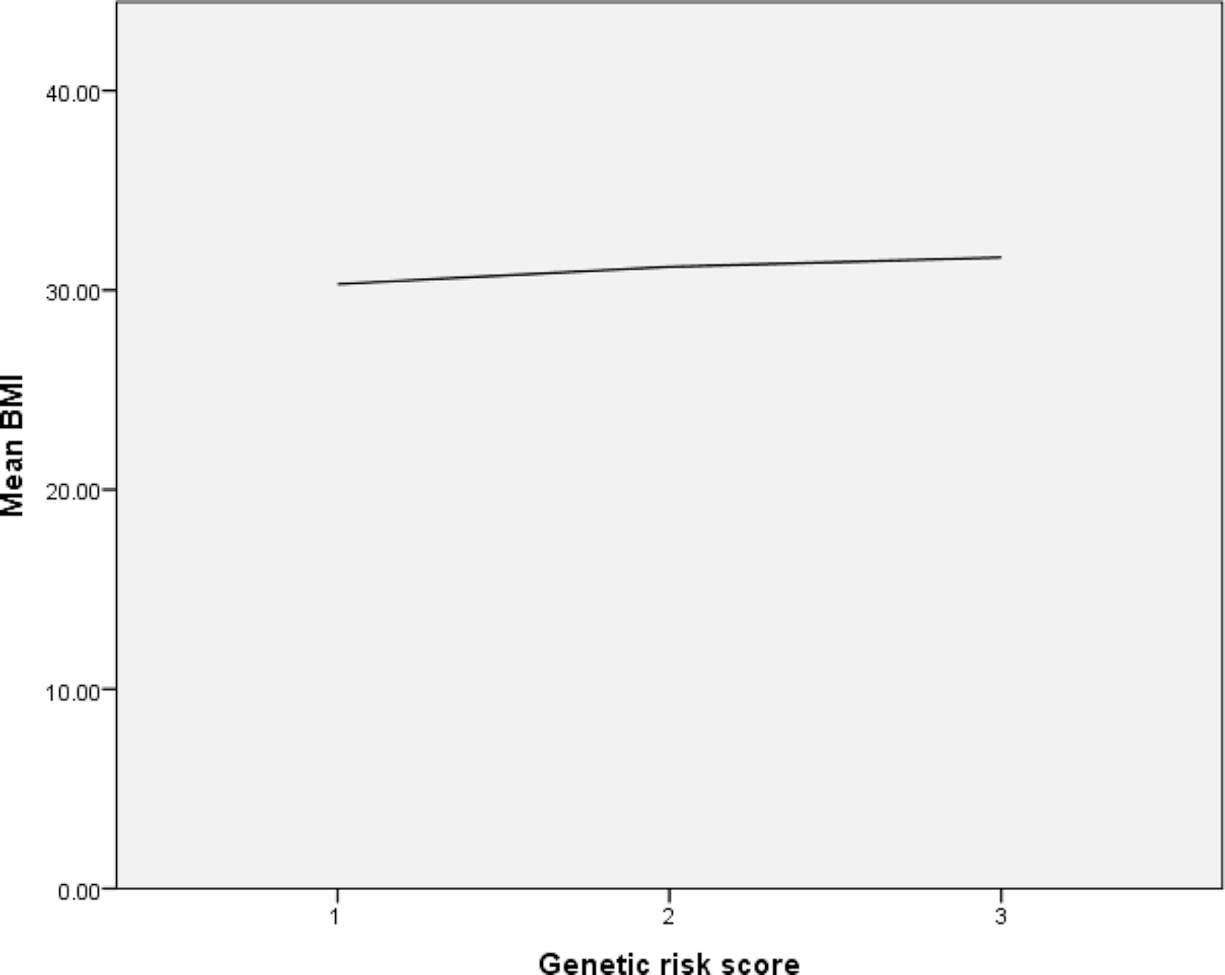
Mean and SD of BMI according to tertiles of GRS

**Fig. 8:**
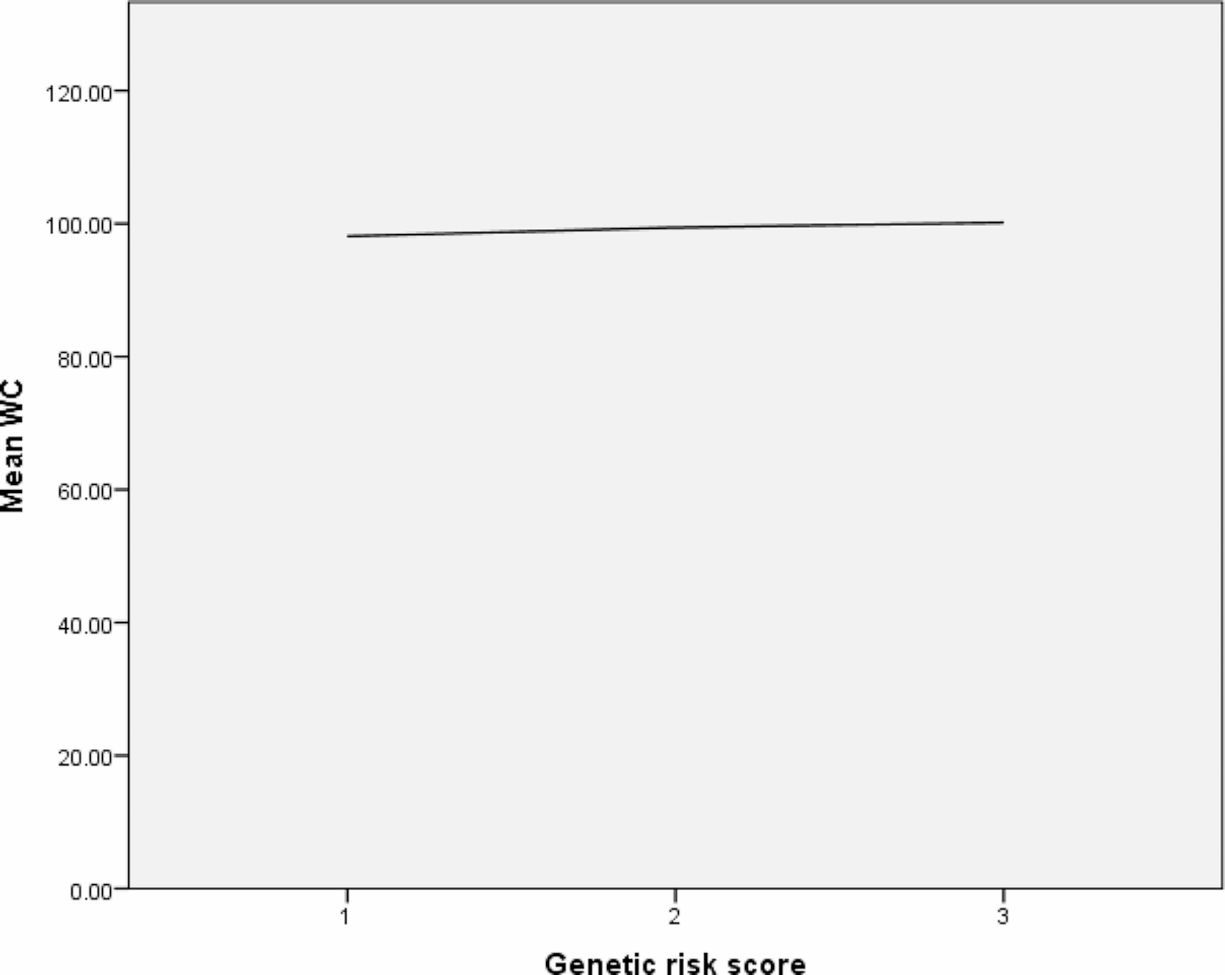
Mean and SD of WC according to tertiles of GRS

**Fig. 9:**
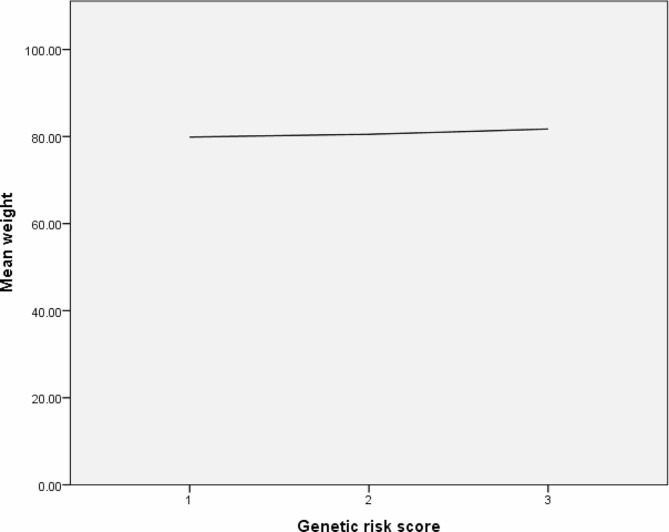
Mean and SD of weight according to tertiles of GRS

**Fig. 10:**
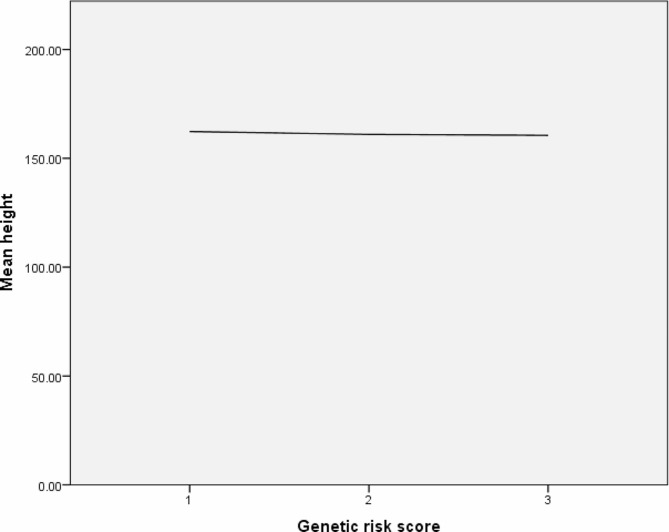
Mean and SD of height according to tertiles of GRS

The baseline characteristics of study participants, categorized according to the GRS, were presented in Table 2; Figs. 1, 2, 3, 4, 5, 6, 7, 8, 9 and 10. As shown in this table, in the crude model, a significant mean difference was observed among tertiles of the GRS in terms of height (*P* = 0.010) and marginally significant for BMI (*P* = 0.051). After controlling for potentially confounding variables (age, energy intake, PA, and BMI), the mean difference of height (*P* = 0.020) remained significant among tertiles of the GRS.

**Table 2:** Mean and SD of general characteristics according to tertiles of GRS in obese and overweight women (*n* = 279)

Variables†	GRS				P-value	P-value ^a^
	Mean ± SD					
	T_1_ (*n* = 114)		T_2_ (*n* = 64)	T_3_ (*n* = 101)		
Age (Y)	35.98 ± 8.74		36.65 ± 8.48	36.94 ± 8.15	0.699	0.902
PA (MET-min/week)	1075.23 ± 1073.96		893.91 ± 996.58	956.47 ± 1169.82	0.57	0.69
**Anthropometry and Body Composition**						
Weight (kg)	80.00 ± 10.32	78.55 ± 11.12		80.90 ± 11.35	0.403	0.77
Height (cm)	162.56 ± 5.51	160.77 ± 6.29		160.27 ± 5.66	**0.010**	0.02
WC (cm)	97.71 ± 9.01	98.05 ± 9.19		99.44 ± 9.73	0.379	0.408
BMI (kg/)	30.22 ± 3.54	30.53 ± 3.44		31.43 ± 4.00	**0.051**	0.182
BF (%)	40.55 ± 4.89	41.79 ± 4.81		41.75 ± 6.00	0.170	0.107
**Blood pressure**						
SBP (mmHg)	110.50 ± 11.88	111.12 ± 15.20		111.98 ± 14.24	0.738	0.869
DBP (mmHg)	77.34 ± 9.74	77.64 ± 10.09		77.76 ± 9.22	0.950	0.766
**Metabolic factors**						
FBS (mg/dl)	87.05 ± 9.04	86.03 ± 7.44		88.34 ± 11.53	0.372	0.695
TG (mg/dl)	122.10 ± 67.92	109.47 ± 51.64		128.08 ± 81.78	0.299	0.306
HDL (mg/dl)	47.04 ± 9.85	48.43 ± 12.41		45.06 ± 9.98	0.167	0.243

BF%; body fat percentage; BMI: body mass index; DBP: diastolic blood pressure; FBS: fasting blood sugar; GRS: genetic risk scores; HDL: high density lipoprotein; PA: physical activity; SD: standard deviation; SBP: systolic blood pressure; T: tertile; TG: triglyceride; WC: waist circumference.

† Calculated by analysis of variance (ANOVA)

a: Adjusted for age, BMI, physical activity, and total energy intake.

*p* < 0.05 was considered significant

### Mean and SD of dietary intake according to tertiles of CSI and N6/N3

Dietary intakes of participants across tertiles of CSI and N6/N3 ratio were presented in (Additional file 1: Table S1). After adjustment with the energy intake, there were significant mean differences of legumes (*P* = 0.049), vegetables(*P* = 0.001), high fat dairy(*P* = 0.001), carbohydrate(*P* = 0.001), total fat(*P* = 0.001), monounsaturated fatty acid (MUFA) (*P* = 0.001), SFA(*P* = 0.001), vitamin C(*P* = 0.001), folate(*P* = 0.001), vitamin B12(*P* = 0.001), total fiber (*P* = 0.001), linolenic acid (*P* = 0.005), potassium (*P* = 0.002), niacin (*P* = 0.002), thiamin (*P* = 0.012), and vitamin B6 (*P* = 0.016) across tertiles of CSI, also a significant mean difference was observed among tertiles of the N6/N3 in terms of MUFA (*P* = 0.034), polyunsaturated fatty acid (PUFA) (*P* = 0.029), linoleic acid (*P* = 0.030), and sodium (*P* = 0.046).

### The interaction between GRS with CSI and N6/N3 ratio on MetS risk factors

The interaction between tertiles of the GRS with tertiles of the CSI and N6/N3 ratio on MetS risk factors were presented in Table 3. In the crude model, a marginally positive interaction was observed between T3 of GRS and T3 of CSI on FBS (β = 7.21, 95%CI= -0.02,14.46, *P* = 0.051), and also a marginally negative interaction was observed between T2 of GRS with T3 of on HDL (β= -8.74, 95%CI= -17.68,0.19, *P* = 0.055), also the interaction between T2 of GRS and T2 of N6/N3 ratio on WC (β = 8.45, 95%CI = 1.33, 15.57, *P* = 0.020), and T3 of GRS and T3 of N6/N3 ratio on TG (β = 62.00, 95%CI = 7.52, 116.48, *P* = 0.026) were positive. After controlling for potential confounders including age, energy intake, PA and BMI in model 1, there was a positive interaction between T2 of GRS and T2 of N6/N3 ratio on WC (β = 7.95, 95%CI = 0.83,15.08, *P* = 0.029), T3 of GRS and T2 of N6/N3 ratio on DBP (β = 5.93, 95%CI= -0.76,12.63, *P* = 0.083), and FBS (β = 6.47, 95%CI = 0.59,13.53, *P* = 0.073), T3 of GRS and T3 of N6/N3 ratio on TG (β = 54.42, 95%CI = 1.76,107.08, *P* = 0.043), and T3 of GRS and T3 of CSI on BF% (β = 3.55, 95%CI= -0.35,7.45, *P* = 0.075). Also, T2 of GRS in the interaction with T3 of CSI leads to an decrease − 8.35 mg/dl in HDL level after adjustment in model 1 (β= -8.35, 95%CI= -17.34,0.62, *P* = 0.068).

**Table 3:** The interaction between GRS with CSI and N6/N3 on metabolic syndrome risk factors in obese and overweight women (*n* = 279)

Variable	GRS	CSI												
		T1	T2						T3					
			Crude			Model 1			Crude			Model 1		
			B	CI	P	B	CI	P	B	CI	P	B	CI	P
**Anthropometric measurements**														
WC (cm)	T1	Reference	Reference						Reference					
	T2		1.67	-5.48, 8.84	0.646	1.60	-5.37, 8.59	0.652	-1.17	-8.66, 6.31	0.758	-0.56	-7.83, 6.70	0.879
	T3		-3.56	-10.41, 3.28	0.307	-4.20	-10.87, 2.46	0.216	-3.44	-10.11, 3.21	0.310	-3.66	-10.16, 2.83	0.269
BF (%)	T1	Reference	Reference						Reference					
	T2		0.54	-3.69, 4.77	0.802	0.77	-3.42, 4.97	0.718	-2.40	-6.83, 2.02	0.287	-1.58	-5.96, 2.78	0.477
	T3		-0.05	-4.10, 3.99	0.978	0.22	-3.78, 4.22	0.913	2.78	-1.16, 6.72	0.167	3.55	-0.35, 7.45	**0.075**
**Blood pressure**														
SBP (mmHg)	T1	Reference	Reference						Reference					
	T2		2.80	-8.47, 14.08	0.626	2.11	-8.60, 12.82	0.699	8.76	-3.00, 20.53	0.144	7.00	-4.11, 18.11	0.217
	T3		1.69	-9.16, 12.54	0.760	0.01	-10.29, 10.33	0.997	-2.47	-12.95, 8.00	0.644	-5.44	-15.38, 4.48	0.283
DBP (mmHg)	T1	Reference	Reference						Reference					
	T2		5.41	-2.63, 13.47	0.188	5.46	-2.29, 13.22	0.167	2.67	-5.72, 11.08	0.532	1.15	-6.89, 9.20	0.778
	T3		3.09	-4.65, 10.85	0.434	2.50	-4.96, 9.97	0.511	-1.89	-9.38, 5.59	0.620	-3.72	-10.91, 3.47	0.311
**Metabolic factors**														
FBS (mg/dl)	T1	Reference	Reference						Reference					
	T2		0.37	-7.45, 8.20	0.926	-0.07	-7.58, 7.43	0.984	2.78	-5.40, 10.96	0.505	1.45	-6.35, 9.25	0.715
	T3		3.02	-4.46, 10.50	0.429	2.81	-4.35, 9.98	0.441	7.21	-0.02, 14.46	**0.051**	5.54	-1.39, 12.47	0.117
TG (mg/dl)	T1	Reference	Reference						Reference					
	T2		-14.93	30.23, -74.19	0.621	-16.32	-73.98, 41.34	0.579	14.49	-47.44, 76.43	0.646	10.54	-49.39, 70.48	0.730
	T3		25.23	28.91, -31.43	0.383	22.15	-32.92, 77.23	0.431	39.92	-14.88, 94.73	0.153	31.13	-22.11, 84.38	0.252
HDL (mg/dl)	T1	Reference	Reference						Reference					
	T2		3.18	-5.36, 11.74	0.465	3.38	-5.25, 12.02	0.443	-8.74	-17.68, 0.19	**0.055**	-8.35	-17.34, 0.62	**0.068**
	T3		2.30	-5.87, 10.48	0.580	2.20	-6.05, 10.45	0.601	-1.56	-9.47, 6.34	0.699	-1.15	-9.13, 6.82	0.777
**Variable**	**GRS**	**N6/N3**												
		**T1**	**T2**						**T3**					
			Crude			Model 1			Crude			Model 1		
			B	CI	P	B	CI	P	B	CI	P	B	CI	P
**Anthropometric measurements**														
WC (cm)	T1	Reference	Reference						Reference					
	T2		8.45	1.33, 15.57	**0.020**	7.95	0.83, 15.08	**0.029**	2.15	-5.39, 9.69	0.576	1.28	-6.29, 8.86	0.740
	T3		1.29	-5.07, 7.66	0.690	1.89	-4.46, 8.25	0.559	0.44	-5.91, 6.79	0.892	0.04	-6.28, 6.37	0.989
BF (%)	T1	Reference												
	T2		3.34	-0.89, 7.58	0.122	3.49	-0.75, 7.74	0.107	1.26	-3.22, 5.75	0.580	1.69	-2.83, 6.21	0.464
	T3		0.88	-2.90, 4.67	0.648	0.76	-3.03, 4.55	0.695	2.25	-1.52, 6.03	0.242	2.42	-1.35, 6.19	0.208
**Blood pressure**														
SBP (mmHg)	T1	Reference	Reference						Reference					
	T2		4.34	-6.45, 15.13	0.431	1.67	-8.78, 12.14	0.754	4.18	-7.28, 15.64	0.475	0.53	-10.53, 11.60	0.925
	T3		0.75	− 0.9.06, 10.57	0.881	1.84	-7.59, 11.28	0.702	-5.31	-15.10, 4.46	0.287	-6.69	-16.06, 2.67	0.161
DBP (mmHg)	T1	Reference												
	T2		4.31	-3.30, 11.93	0.267	3.40	-4.02, 10.82	0.369	1.61	-6.47, 9.70	0.696	-0.56	-8.41, 7.29	0.888
	T3		5.28	-1.64, 12.21	0.135	5.93	-0.76, 12.63	**0.083**	0.73	-6.16, 7.64	0.834	-0.01	-6.66, 6.62	0.995
**Metabolic factors**														
FBS (mg/dl)	T1	Reference	Reference						Reference					
	T2		2.75	-5.30, 10.81	0.503	0.48	-7.18, 8.16	0.901	3.71	-4.72, 12.14	0.389	1.34	-6.61, 9.30	0.740
	T3		5.54	-1.94, 13.03	0.147	6.47	-0.59, 13.53	**0.073**	5.15	-1.99, 12.30	0.158	3.80	-2.92, 10.52	0.268
TG (mg/dl)	T1	Reference	Reference						Reference					
	T2		10.52	-50.88, 71.93	0.737	-4.88	-64.99, 55.21	0.873	29.26	-34.98, 93.51	0.372	13.21	-49.11, 75.54	0.678
	T3		14.69	-42.37, 71.76	0.614	22.86	-32.48, 78.20	0.418	62.00	7.52, 116.48	**0.026**	54.42	1.76, 107.08	**0.043**
HDL (mg/dl)	T1	Reference	Reference						Reference					
	T2		-3.42	-12.31, 5.47	0.451	-3.25	-12.29, 5.78	0.481	-7.10	-16.41, 2.20	0.135	-6.85	-16.23, 2.51	0.152
	T3		-1.55	-9.81, 6.71	0.713	-1.49	-9.82, 6.82	0.725	-2.73	-10.63, 5.15	0.497	-2.42	-10.34, 5.50	0.549

BF%; body fat percentage; BMI: body mass index; CI: confidence interval; CSI: cholesterol to saturated fat index; DBP: diastolic blood pressure; FBS: fasting blood sugar; GRS: genetic risk scores; HDL: high-density lipoprotein; SD: standard deviation; SBP: systolic blood pressure; T: tertile; TG: triglyceride; WC: waist circumference.

GLM was performed to identify the interaction between GRS with CSI and N6/N3 on metabolic syndrome risk factors.

model 1 = adjusted for potential confounding factors including (age, energy intake, physical activity and BMI).

*p* < 0.1 was considered significant

## Discussion

In the current cross-sectional study, we investigated the interaction between GRS and fatty acid quality indices with MetS among 279 overweight and obese women. Accoring to findings, after controlling for potential confounders, we observed that the interaction of GRS and N6/N3 has a positive significant association with WC, DBP, FBS and TG. Also, the interaction of GRS and CSI had a positive significant association with BF%. Moreover, the interaction of GRS and CSI had a negative significant association with HDL.

The etiology of MetS is complicated, however documents have indicated that dietary patterns, physical activity level and genetic polymorphism take a part in its pathogenesis [10, 44]. Moreover, Asians are more likely prone to develop MetS than non-Asians, Europeans and Americans [45]. Individual SNPs have been studied as a useful genetic tool to predict the tendency to MetS or obesity in different age groups [46]. GRS as an non-modifiable factor have been recognized as an associated factor for obesity, MetS, and type 2 diabetes in previous studies [25, 47, 48]. On the other hand, dietary patterns which contain too much fried foods, soda and meat can increase the risk of MetS [12, 49]; however, other dietary ingrediants such as fruits and vegetables can have a protective role against MetS and other chronic diseases [10].

### Findings on FBS and anthropometric indices

The findings on interaction between GRS and dietary patterns especially different dietary quality indices with MetS incidence is rare and unclear. In a prospective nested case-control study among 1196 diabetic and 1337 nondiabetic men, the highest risk of type 2 diabtes in relationship with a western dietary pattern was belonged to the highest GRS tertile [50]. Our findings revealed that the interaction of the highest tertile of GRS with the N6/N3 positively was associated with FBS level. A study which conducted among both men and women found that increasing ratio of saturated fat to carbohydrate related to higher HOMA levels in minor allele carriers (PLIN11482G > A) [51].

According to our findings, the interaction of GRS and N6/N3 and the interaction of GRS and CSI were positively associated with WC and BF%, respectively. This finding suggest that dietary fatty acid amouts and composition may potentially influence on genetic susceptibility of being obese [52, 53]. Findings from 18 cohorts of European ancestry found that GRS and diet may increase risk of obesity [54]. Morover, a cross-sectional study among 476 Iranian participants assessed the interaction of a high fat and sugar intake with a SNP of vascular endothelial growth factor (rs10738760), and revealed a increased risk of MetS [55]. A study among Ghanaian population revealed an interaction between 4-SNP GRS and fat intake on WC which are associated with higher amounts of mortality [56]. Studies in 354 Spanish children and adolscents, 1754 French individulas and 2163 American participants have shown a significant interaction of FTO SNP rs9939609 with MUFAs and SFAs on BMI [57–59]. Previous studies among 28,449 individuals in Malmo [60] and 2163 individulas in United States of America have indicated significant intractions of the FTO SNP rs9939609 and fat intake on BMI [57], however, a meta-analysis on 177,330 participants failed to identify this intraction [52]. High SFA intake presented significantly a higher BMI in the GG carriers than in A carriers [61]. On the other hand, low PUFA intake revealed an inverse association with risk of BMI of more than 30 kg/m^2^ in the presence of ADAM17i33708A polymorphism among 936 men amd women [62]. Overally, conflicting evidence in many previous studies regarding the effect of fat intake and obesity-related parameters could be because of the gene-diet interactions and genetic heterogeneity across various ethnic groups [63, 64]. Hence, the synergism effect of genetic and dietay intakes should be considered in future studies. According to findings of a parallel controlled-feeding trial, the mechanisms which can explain the increase in anthropometric indices by adhereing a high SFA diet including as increase in the expression of inflammatory genes in adipose tissue, and decrease in the expression of genes involved in fatty acid β-oxidation and synthesis of TG [65].

### Findings on lipid profile indices

A study in 1680 South Asians has demonstrated a significant interaction of fat intake with the risk allele ‘T’ of the TCF7L2 SNP rs 12,255,372 on HDL [66]. In a population-based study findings showed that the women who carries the A allele of APOA1 gene (G-A polymorphism) had higher HDL in response of high PUFA intake [67]. While, in our study the interaction of GRS and CSI showed a negative significant association with HDL. Accoring to our findings, after controlling for potential confounders, we observed that the interaction of GRS and N6/N3 has a positive significant association with TG. In a population-based study, the interaction of PUFA intake and PPARAL162V revealed a lower TG level with higher PUFA intake in the V carries [68]. The mechanism which is related to the alteration of lipid profile maybe because of altering the lipoprotein lipase activity in adipose tissue and muscles and decreasing energy expenditure [69, 70].

An eleven year longitudinal study revealed that the western dietary pattern increased MetS risk among GRS tertiles in Whites participants with age range of 45–64 years [28]. However, in the mentioned study, high-fat dairy pattern showed a protective effect against MetS especially in the lowest GRS tertile [28]. There are several studies which have shown a protective role of high-fat dairy products on MetS and type 2 diabetes [11, 71], but deleterious effect of western dietary pattern is related to red and processed meat, fried foods and sweets [28]. Also, Hardy et al. found that FAD1 and FAD2 genes were linked to rs174548 which is a SNP in the GRS [28]. FAD1 and FAD2 genes involves in long chain polyunsaturated fatty acid synthesis and are linked with CVD and other health outcomes [72]. Different fatty acids indicate various effect on metabolic outcomes, for instance conjugated linoleic acid has been observed to decrease insulin resistance and inflammation, while arachidonic acid has indicated pro-inflammatory condition and has increased atherosclerotic damage [72]. High ratio of arachidonic acid to linoleic acid among individuals who carrying FAD may be detrimental due to more susceptibility to inflammatory conditions [73]. Overally, various findings of different studies could be due to the different age, gender, population, continent, or specific criteria to define MetS and other related outcomes. Moreover, analyzing the genetic associations with main outcomes in Iranian population may not be ideal, because of differences between risk allele frequency of the Iranian population and the other poulations [74, 75]. These inconsistencie maybe due to the variation in the genetic architecture between different ancestries [76].

To the best of authors knowledge, this is the first study that investigated the interaction between GRS and fatty acid quality indices with MetS among overweight and obese women. Also, our study population was highly homogeneous, because it conducted only among Iranian subjects. However, findings cannot be applied to reveal cause and effect regarding the cross-sectional type of the study. Also, using FFQ to assess dietary intake is one of limitations due to its recall bias. Moreover, dietary intakes can vary by socioeconomic status and culture; although we adjusted findings to several confounders, remain effect of these factors may impact on results. Thus, it is suggested to replicate the study in other large populations.

## Conclusion

As theses days, MetS, obesity and other non-communicable diseases occur in a wide range, it is fundamental to develop health prevention programs which help to detection, early diagnosis and treatment of MetS. It seems the interaction of GRS and fatty acid quality indices is positively associated with several components of metabolic syndrome such as WC, TG and BF%. However, more studies with larger sample size are needed to confirm these findings.

### Electronic supplementary material

Below is the link to the electronic supplementary material.

Supplementary Material 1

## Data Availability

The datasets analysed during the current study are not publicly available due ethical issues but are available from the corresponding author on reasonable request.

## Abbreviations

CAV: Caveolin
CRY: Cryptochrome
CVD: Cardiovascular disease
CSI: Cholesterol-Saturated Fat Index
EFA: Essential fatty acids
GRS: Genetic risk score
GWAS: Genome-wide association studies
HDL: High-density lipoprotein
MC4R: Melanocortin-4 Receptor
PA: Physical Activity
SFA: Saturated fatty acid
SNP: Single nucleotide polymorphisms
T2DM: Type 2 diabetes mellitus
