# Supplementary material for: Investigation of the interaction between genetic risk score (GRS) and fatty acid quality indices on metabolic syndrome among overweight and obese women
Source: BMC Med Genomics. 2024 Apr 29;17:113. doi: 10.1186/s12920-024-01838-2 (PMC11057091; doi:10.1186/s12920-024-01838-2)
Supplement: Supplementary file 1 — Supplementary Material 1 [file 12920_2024_1838_MOESM1_ESM.docx]

**Table S1:** Mean and SD of dietary intake according to tertiles of CSI and N6/N3 in obese and overweight women (n=279).

| **Variables**† | **CSI** | | | |
| --- | --- | --- | --- | --- |
|  | **Mean±SD** | | | **p-value** |
|  | **T_1_ (n=78)** | **T_2_ (n=79)** | **T_3_ (n=78)** |  |
| **Food group** |  | | |  |
| Grains (g/d) | 534.37±254.96 | 483.62±230.34 | 386.84±175.35 | 0.18 |
| Nuts (g/d) | 0.65±0.38 | 0.53±0.42 | 0.35±0.38 | 0.401 |
| Legumes (g/d) | 48.56±28.88 | 56.19±46.18 | 49.64±48.69 | **0.049** |
| Vegetables (g/d) | 426.14±261.87 | 497.34±277.79 | 372.20±246.70 | **0.001** |
| High fat dairy (ml/d) | 186.45±197.52 | 80.47±92.85 | 38.19±51.94 | **0.001** |
| Low-fat dairy (ml/d) | 316.98±271.23 | 301.54±198.88 | 226.38±171.83 | 0.556 |
| Fish (g/d) | 12.06±11.83 | 10.99±13.42 | 11.15±9.36 | 0.525 |
| Poultry (g/d) | 38.34±47.19 | 30.18±20.27 | 33.10±32.42 | 0.137 |
| Egg (g/d) | 22.89±15.16 | 20.06±12.72 | 21.43±13.38 | 0.172 |
| Fast food (g/d) | 26.78±34.80 | 14.61±19.07 | 14.92±19.55 | 0.111 |
| Red meat (g/d) | 30.78±22.78 | 21.41±15.91 | 14.62±14.22 | 0.122 |
| **Nutrient intake** |  | | | |
| Energy (kcal/d) | 3122.26±695.37 | 2597.39±587.89 | 2022.86±494.39 | - |
| Protein (g/d) | 103.59±29.31 | 88.24±20.17 | 71.25±23.35 | 0.889 |
| Carbohydrate (g/d) | 429.78±121.87 | 375.94±109.52 | 298.39±88.75 | **0.001** |
| Total fat (g/d) | 119.57±29.78 | 91.94±22.66 | 66.87±17.22 | **0.001** |
| MUFA (g/d) | 38.67±10.57 | 30.61±8.39 | 22.48±6.66 | **0.001** |
| PUFA (g/d) | 23.26±9.44 | 20.32±7.95 | 15.35±5.54 | 0.138 |
| SFA (mg/d) | 38.75±10.94 | 26.00±4.59 | 18.31±4.37 | **0.001** |
| Trans fat | 0.001±0.002 | 0.001±0.002 | 0.001±0.003 | 0.674 |
| Linolenic acid (g/d) | 1.53±0.61 | 1.26±0.63 | 0.87±0.41 | **0.005** |
| Linoleic acid (g/d) | 20.12±9.16 | 17.65±7.60 | 13.13±5.27 | 0.127 |
| EPA (g/d) | 0.03±0.03 | 0.03±0.04 | 0.03±0.03 | 0.768 |
| DHA (g/d) | 0.11±0.11 | 0.10±0.13 | 0.10±0.09 | 0.745 |
| Sodium (mg/d) | 4861.72±1543.02 | 4194.65±1236.91 | 3530.78±1091.45 | 0.977 |
| Potassium (mEq/d) | 4927.99±1614.83 | 4571.40±1411.98 | 3460.41±1338.79 | **0.002** |
| Calcium (mg/d) | 1396.02±438.93 | 1171.16±323.52 | 886.61±310.26 | 0.081 |
| Vitamin C (mg/d) | 212.34±119.00 | 206.84±157.74 | 165.00±102.33 | **0.001** |
| Vitamin E (mg/d) | 18.95±9.18 | 17.87±9.19 | 13.83±6.48 | 0.309 |
| Vitamin D (ug/d) | 2.31±1.95 | 1.98±1.32 | 1.56±1.26 | 0.754 |
| Thiamin (mg/d) | 2.36±0.62 | 2.07±0.56 | 1.68±0.52 | **0.012** |
| Riboflavin (mg/d) | 2.64±0.85 | 2.20±0.73 | 1.70±0.55 | 0.606 |
| Niacin (mg/d) | 28.56±10.63 | 24.71±6.51 | 21.09±6.93 | **0.002** |
| Vitamin B6 (mg/d) | 2.46±0.76 | 2.22±0.59 | 1.74±0.57 | **0.016** |
| Folate (mcg/d) | 656.95±172.73 | 624.48±157.98 | 519.93±161.77 | **0.001** |
| Vitamin B12 (mcg/d) | 5.76±2.92 | 4.04±1.38 | 3.24±1.43 | **0.001** |
| Total fiber (g/d) | 47.51±18.51 | 46.72±18.32 | 39.38±17.43 | **0.001** |
| **Variables**† | **N6/N3** | | |  |
|  | **Mean±SD** | | | **P-value^*^** |
|  | **T1(n=93)** | **T2(n=93)** | **T3(n=93)** |  |
| **Food group** |  |  |  |  |
| Grains (g/d) | 626.30±257.50 | 455.01±194.21 | 343.78±116.82 | 0.80 |
| Nuts (g/d) | 0.67±0.38 | 0.53±0.40 | 0.30±0.34 | 0.337 |
| Legumes (g/d) | 55.22±42.12 | 54.65±45.02 | 38.39±32.47 | 0.216 |
| Vegetables (g/d) | 504.04±277.69 | 455.85±271.89 | 316.23±189.67 | 0.094 |
| High fat dairy (ml/d) | 163.05±183.00 | 97.79±121.75 | 42.75±55.11 | 0.298 |
| Low-fat dairy (ml/d) | 346.30±277.60 | 282.90±204.63 | 223.62±150.88 | 0.771 |
| Fish (g/d) | 13.75±15.65 | 11.24±11.07 | 9.36±8.81 | 0.996 |
| Poultry (g/d) | 45.60±55.96 | 31.70±29.99 | 28.12±23.10 | 0.328 |
| Egg (g/d) | 25.27±17.06 | 22.53±13.63 | 17.38±10.67 | 0.385 |
| Fast food (g/d) | 27.02±34.16 | 17.00±20.52 | 13.62±16.33 | 0.710 |
| Red meat (g/d) | 31.64±20.16 | 20.75±19.16 | 12.47±8.39 | 0.058 |
| **Nutrient intake** |  | | | |
| Energy (kcal/d) | 3468.26±402.67 | 2545.52±190.36 | 1799.81±271.01 | **-** |
| Protein (g/d) | 114.98±24.09 | 87.51±17.49 | 63.37±13.30 | 0.584 |
| Carbohydrate (g/d) | 502.95±82.83 | 353.96±47.13 | 255.92±53.31 | 0.099 |
| Total fat (g/d) | 122.50±27.88 | 95.28±20.53 | 63.74±15.19 | 0.096 |
| Cholesterol (g/d) | 326.34±123.41 | 241.50±75.48 | 189.51±56.43 | 0.438 |
| MUFA (g/d) | 39.10±9.87 | 32.22±9.23 | 21.80±6.55 | **0.034** |
| PUFA (g/d) | 24.25±7.54 | 21.12±8.80 | 14.24±5.48 | **0.029** |
| SFA (mg/d) | 37.54±11.27 | 27.37±6.58 | 18.86±5.14 | 0.385 |
| Trans fat | 0.001±0.002 | 0.001±0.002 | 0.001±0.003 | 0.608 |
| Linolenic acid (g/d) | 1.58±0.55 | 1.26±0.67 | 0.82±0.40 | 0.073 |
| Linoleic acid (g/d) | 20.80±7.42 | 18.44±8.59 | 12.27±5.34 | **0.030** |
| EPA (g/d) | 0.03±0.04 | 0.03±0.04 | 0.02±0.02 | 0.833 |
| DHA (g/d) | 0.12±0.13 | 0.10±0.12 | 0.08±0.08 | 0.948 |
| Sodium (mg/d) | 5279.14±1367.51 | 4106.78±1175.76 | 3327.17±1012.63 | **0.046** |
| Potassium (mEq/d) | 5702.71±1228.83 | 4259.35±1097.22 | 2983.18±898.34 | 0.292 |
| Calcium (mg/d) | 1508.44±363.20 | 1153.69±321.05 | 822.09±238.20 | 0.081 |
| Vitamin C (mg/d) | 267.71±143.24 | 189.35±106.95 | 126.66±69.58 | 0.507 |
| Vitamin E (mg/d) | 20.03±9.20 | 18.25±9.65 | 13.39±7.21 | 0.212 |
| Vitamin D (ug/d) | 2.55±2.08 | 1.91±1.41 | 1.43±1.03 | 0.983 |
| Thiamin (mg/d) | 2.71±0.49 | 2.03±0.38 | 1.47±0.32 | 0.960 |
| Riboflavin (mg/d) | 2.90±0.78 | 2.14±0.53 | 1.53±0.37 | 0.516 |
| Niacin (mg/d) | 33.23±9.48 | 24.09±5.16 | 18.06±4.17 | 0.467 |
| Vitamin B6 (mg/d) | 2.82±0.58 | 2.10±0.45 | 1.53±0.34 | 0.239 |
| Folate (mcg/d) | 768.05±132.93 | 592.38±110.35 | 452.14±110.36 | 0.960 |
| Vitamin B12 (mcg/d) | 5.85±3.02 | 4.03±1.71 | 3.16±1.38 | 0.452 |
| Total fiber (g/d) | 58.99±18.26 | 44.74±14.61 | 31.87±12.65 | 0.835 |
| CSI: Cholesterol to saturated fat index; DHA: docosahexaenoic acid; EPA: eicosapentaenoic acid; MUFA; monounsaturated fatty acid; PUFA: polyunsaturated fatty acid; SFA: saturated fatty acid; T: tertile.  Data are mean ± SD  P-value*: ANCOVA was performed to adjust the potential confounding factor (energy intake).  p < 0.05 was considered significant. | | | | |
